# Impact of ovarian hyperstimulation syndrome on intracytoplasmic sperm injection outcomes in poly-cystic ovarian syndrome women: A cross-sectional study

**DOI:** 10.18502/ijrm.v23i2.18496

**Published:** 2025-05-01

**Authors:** Mufeda Ali Jwad, Maryam Hussein Khaleefah, Ramih Abd AlFattah Naser

**Affiliations:** ^1^Department of Physiology, High Institute for Infertility Diagnosis and Assisted Reproductive Technologies, Al-Nahrain University, Baghdad, Iraq.; ^2^Department of Applied Embryology, High Institute for Infertility Diagnosis and Assisted Reproductive Technologies, Al-Nahrain University, Baghdad, Iraq.

**Keywords:** Polycystic ovary syndrome, Ovarian hyperstimulation syndrome, Intracytoplasmic sperm injection, Ovulation induction.

## Abstract

**Background:**

Polycystic ovarian syndrome (PCOS) is a complex disorder that affects the endocrine, metabolic, and reproductive systems. While controlled ovarian hyperstimulation can increase the number of oocytes retrieved and improve the number of good-quality embryos, it may also negatively affect oocyte maturity, embryo quality, endometrial receptivity, and pregnancy outcomes.

**Objective:**

This study aimed to find out if ovarian hyperstimulation syndrome (OHSS) has negative effects on intracytoplasmic sperm injection outcomes in a sample of PCOS women.

**Materials and Methods:**

In this cross-sectional study, data of 84 PCOS women who underwent controlled ovarian stimulation using a flexible antagonist protocol-treated intracytoplasmic sperm injection cycles referred to the Infertility Department of Higher Institute for the Diagnosis of Infertility and Assisted Reproduction Techniques, Baghdad, Iraq between January 2020 and December 2023 was extracted from their medical records. Participants were categorized into 2 groups after undergoing controlled ovarian hyperstimulation protocol: PCOS with OHSS (n = 54) and PCOS without OHSS (n = 30). The dosage was tailored based on age, body mass index, and response to previous stimulation cycles, if applicable. The trigger was administered using decapeptyl 0.2 mg alone or in combination with recombinant human chorionic gonadotrophin, depending on the estradiol levels at the time of the trigger.

**Results:**

Significant differences were observed in the duration of stimulation (p = 0.005), oocyte yield (p = 0.001), mature oocytes (p = 0.001), and fertilized oocytes (p = 0.036); however, no significant difference was observed, neither in number of good quality embryos (p = 0.52) nor in pregnancy rate (p = 0.54) after fresh and frozen embryo transfer between the 2 groups.

**Conclusion:**

OHSS does not affect the embryo quality or pregnancy rate in PCOS women.

## 1. Introduction

Reproduction results from coordination between the brain, gonads, and different body parts (1). Polycystic ovarian syndrome can interfere with women fertility (2), which may have metabolic and reproductive drawbacks that can be seen in both reproductive and post-menopausal periods (3). According to Rotterdam criteria, polycystic ovarian syndrome (PCOS) women have 2 of the following features: “clinical and/or biochemical hyperandrogenism, ovarian dysfunction, and PCOS morphology” (4, 5). Long-term complications such as endometrial carcinoma may develop (6).

The large number of follicles in PCOS may lead to folliculogenesis abnormalities that may result from intra-ovarian hyperandrogenism and hyperinsulinemia (7, 8). Therefore, lower number of mature oocytes can be obtained and as a result bad quality embryos with less developmental potential and lower pregnancy rate (9). In assisted reproductive technologies (ART), controlled ovarian hyperstimulation leads to retrieval of multiple oocytes so that multiple embryos are available for transfer, thereby increasing the efficacy of ART (10). However, at the same time, a higher risk of ovarian hyperstimulation syndrome (OHSS) results (11), which can be classified into mild, moderate, severe, and critical (12). In contrast, in severe cases, a critical condition may develop that can be associated with life-threatening complications such as pulmonary embolism, deep vein thrombosis, and acute renal failure (13). The pathophysiology of OHSS is partly related to vascular endothelial growth factor which has its receptors on the surface of vascular endothelial cells. The vessels become more permeable shifting the fluid to the third space. In addition, it was found that abnormal apoptosis of granulosa cells may have a part in the pathophysiology of the condition but the molecular process behind this is unknown. High androgen level in PCOS leads to atresia of the growing follicles and these are eliminated without tissue injury or inflammation suggesting that this process is a result of programmed cell death (14), in addition to the possible effects of other factors like insulin-like growth factor angiotensin II, epidermal growth factor, transforming growth factor alpha, and others (15).

The primary risk factors for OHSS are PCOS, low body mass index (BMI), young women, and a high antral follicle count ≥ 24. Other risk factors include a history of allergies, high levels of anti-Mullerian hormone > 3.36 ng/mL, high gonadotropins doses, and high serum estradiol (E2), while the secondary risk factors depend on ovarian response to controlled ovarian hyperstimulation (16). Elevated ovarian response, history of OHSS, PCOS (17), small follicle count ( > 14 follicles with a diameter of 11 mm), a large number of retrieved oocytes ( > 20), and elevated E2 levels > 2500 pg/ml, have been implicated as potential etiological factors. This is because when the E2 is high, fluid extravasation increase due to higher level of chemical mediators or precursors that augment this process in addition to increase capillary permeability. Thus, OHSS may develop (18). However, none alone is enough for the detection of OHSS (19).

The presence of OHSS was thought to have a detrimental effect on ART results so this study aimed to find out if OHSS has negative effects on intracytoplasmic sperm injection (ICSI) outcomes in a sample of PCOS women.

## 2. Materials and Methods 

### Study design

In this cross-sectional study, data of 84 PCOS women who referred to the Infertility Department of Higher Institute for the Diagnosis of Infertility and Assisted Reproduction Techniques, Baghdad, Iraq between January 2020 and December 2023 were extracted from their medical records and analyzed.

Data of history, general and gynaecological examination, hormonal levels, ovarian stimulation protocol, and ICSI outcome for all participants were extracted from their medical records. After ovarian stimulation the participants were categorized depending on developing signs and symptoms of OHSS into 2 groups: PCOS with OHSS (n = 54) and PCOS without OHSS (n = 30).

The inclusion criteria were PCOS women who met at least 2 of the 3 Rotterdam criteria (clinical and/or biochemical hyperandrogenism, ovarian dysfunction, and PCOS morphology) (4), with or without tubal factors (diagnosed by hysterosalpingogram, hysterosalpingio- contrast sonography, or laparoscopy) or mild male factor infertility (according to partners' seminal fluid analysis). Women with endometriosis (diagnosed by laparoscopy), previous ovarian surgery or surgically removed ovary, normal or poor ovarian reserve according to follicle stimulating hormone (FSH), anti-Mullerian hormone, and antral follicle count, hyperprolactinemia, thyroid disorders, chronic diseases (such as diabetes mellitus, hypertension, and chronic renal disease), pelvic inflammatory disease, age > 40, and a male partner with azoospermia or severe oligo-astheno-teratospermia were excluded from the study.

The diagnosis of OHSS was determined using the following criteria: more than 14 growing follicles with a diameter of 11 mm on the trigger day, retrieval of over 20 oocytes, and an E2 level exceeding 2500 pg/mL on the trigger day.

The included women were monitored for the number of growing follicles on the day of triggering, E2 level, and the number of oocytes retrieved. According to the results obtained, they were classified into 2 groups:

- PCOS without OHSS: 30 PCOS women who did not have signs of OHSS (number of growing follicles on day of trigger < 14 with diameter of 11 mm, < 20 oocytes were retrieved, and E2 level was < 2500 pg/mL).

- PCOS with OHSS: 54 PCOS women who had signs of OHSS (number of growing follicles on day of trigger > 14 with diameter of 11 mm, > 20 oocytes were retrieved and E2 level was > 2500 pg/mL).

### Sample size

The sample size was calculated according to Andrew Fisher's formula. The sample size was estimated to be a minimum of 80 participants by considering a 95% confidence level, a power of 80%.


$$samplesize={\left(\text{z}\text{score}\right)}^{\wedge }2\times \left(\text{std}\text{Dev}\right)\times \left(1-\text{std}\text{Dev}\right)/{\left(\text{Confidence}\text{interval}\right)}^{\wedge }2$$

### Ovarian stimulation

The data relating to the ovarian stimulation process were extracted from the participant's medical records. PCOS women under a flexible antagonist protocol for ICSI cycles were included in the study. All women had a baseline hormonal assay on the 2 ${ߐ}^{\text{nd}}$ or 3 ${ߐ}^{\text{rd}}$ day of the stimulation cycle (FSH, Luteinizing hormone [LH], E2) by enzyme-linked fluorescence assay using Mini vidas analytical equipment (BIOMRIEUX/Italy). For PCOS women, because of their safety, the starting dose of gonadotropin-releasing hormone (GnRH) was determined depending on age, BMI, baseline FSH level, and previous response to ovarian stimulation in the range of 75–150 units per day (flexible antagonist protocol). Transvaginal ultrasound controlled ovarian stimulation, and E2 and LH were assessed when needed.

On the mid-follicular phase, a trans-vaginal ultrasound assessment was performed to start the GnRH antagonist when 3 leading follicles reached 12–13 mm in diameter, with re-scanning every 2–3 days in addition to serial serum E2 level until trigger day (when ≥ 3 leading follicles reach size of ≥ 17 mm) by using triptorelin acetate 0.2 mg with recombinant human chorionic gonadotropin 5000 IU (dual trigger) or triptorelin acetate 0.2 mg alone if E2 level was > 3000 pg/ml. The transvaginal ultrasound-guided oocyte aspiration retrieved the oocytes 34–36 hr after trigger. A single-lumen needle (GyneticsⓇ, Belgium) was used for oocyte retrieval.

### ICSI program

The aspirated follicular fluid samples were immediately sent to an embryologist to collect the cumulus-oocyte complexes. After denudation, the nuclear maturity of the oocytes was proved by the presence of a germinal vesicle (GV) or extruded first polar body in the perivitelline space (Metaphase II [MII]) in addition to the detection of any oocyte abnormality if present. ICSI was conducted on morphologically normal, fully developed MII oocytes only. The oocytes that had been injected with normal sperms were examined for detection of fertilization through the presence of 2 pronucei and 2 polar bodies 16–17 hr after ICSI. The determination of embryo quality was done. Embryos with stage-specific cell numbers, equal size blastomeres, and 10–20% fragmentation were considered as good quality (grade I and II), while those with abnormal cell numbers, unequal size blastomeres, and with > 20% fragmentations were considered as bad quality (20). The fertilization rate was calculated by dividing the number of zygotes/ by the number of injected mature MII oocytes × 100%.

One or 2 embryos were selected for transfer depending on the women's age, embryo quality, and history of previous ICSI cycles. Luteal support was done by progesterone depot 250 mg injection (Bayer, Berlin, Germany) twice weekly and vaginal progesterone (CyclogestⓇ400 mg; Actavis, UK), or (CrinoneⓇ 8% progesterone gel, MERK, Switzerland), and continued daily. The supplementation was started on the day of oocyte retrieval. An assay of serum beta human chorionic gonadotropin was performed on day 14 following embryo transfer.

### Outcome measurements

These included gonadotrophin dose, duration of stimulation, oocytes yield and quality, fertilization rate, embryo yield and quality, and pregnancy rate.

### Ethical Considerations

The study was approved by the Ethics Committee of High Institute for Infertility Diagnosis and Assisted Reproductive Technologies, Al-Nahrain University, Baghdad, Iraq (Code: 0701-PF-2020M26). All participant data collected during this study will remain confidential and will only be used for research purposes. No personally identifiable information will be published or shared without the participant's explicit consent.

### Statistical Analysis

After data collection, they were coded, tabulated, and statistically analyzed using Microsoft Excel 2016 and Statistical Package for Social Sciences Statistics for Windows, Version 28.0. Armonk, NY: IBM Corp was used. Data are presented as mean ± standard deviation. The groups were compared by the nonparametric *t* test (Mann-Whitney test) and Chi-square test. The significance level was assumed at p < 0.05.

## 3. Results

Out of 100 women with PCOS, 16 women were excluded due to stopping the protocol or not doing the oocyte retrieval procedure because of empty follicles or not making fresh or frozen embryo transfer.

Finally, data of 84 women with PCOS who underwent controlled ovarian stimulation with a flexible antagonist protocol were analyzed and compared in 2 groups: OHSS (n = 54) and non-OHSS (n = 30).

There were no significant differences between the groups regarding age, BMI, and duration of infertility (p = 0.91, 0.37, and 0.34, respectively). However, the 2 groups had a significant difference regarding the type of infertility. In the hormonal assessment between the studied groups, no significant differences were observed in the levels of early follicular phase hormones (FSH, LH, E2) (p = 0.72, p = 0.94, p = 0.65). However, the 2 groups showed a significant difference (p = 0.03) in E2 levels on the trigger day (Table I).

Also, there were no significant differences recorded between the 2 groups related to gonadotrophin dose (p = 0.25) and the number of dominant follicles measured by ultrasound (p = 0.14). However, the duration of stimulation was significantly shorter in the OHSS group (p = 0.005), as indicated in table II.

The total number of retrieved and MII oocytes were significantly higher in the OHSS group compared to the non-OHSS group (p = 0.001 and p = 0.0001, respectively). A significantly higher number of zygotes were observed in PCOS women with OHSS; however, no significant differences were observed for the number of MI, GV, abnormal oocytes, or the fertilization rate (%) between the studied groups (Table III).

An assessment of embryo grading was done on day 3 after ICSI. No significant difference (p > 0.05) was observed in the number of embryos when comparing different grades between the 2 groups, as shown in table IV.

A comparison of the clinical pregnancy rates between the 2 groups was done for fresh and frozen cycles and recorded no significant difference (p = 0.10 and p = 0.32, respectively) between the groups (Table V).

**Table 1:** Comparison of demographic characteristics and hormonal levels

**Variables**		**PCOS without OHSS (n = 30)**	**PCOS with OHSS (n = 54)**	**P-value**
Age (yr)*		27.27 ± 0.91 (28.00, 6.25)	30.02 ± 0.68 (30.00, 6.00)	0.91
BMI (kg/m^2^)*		28.07 ± 0.53 (28.60, 4.07)	28.24 ± 0.49 (28.7, 3.7)	0.37
Duration of infertility (yr)*		6.10 ± 0.47 (6.00, 4.00)	6.72 ± 0.41 (7.00, 5.00)	0.34
Type of infertility**				
	**Primary**	19 (63.33)	36 (66.66)	0.0003
	**Secondary**	11 (36.66)	18 (33.33)	0.001
	**P-value**	0.0001	0.0001	—
FSH*		5.25 ± 0.22 (4.92, 1.62)	5.14 ± 0.18 (4.91, 0.18)	0.72
LH*		7.88 ± 0.53 (7.15, 3.69)	8.20 ± 0.43 (7.99, 0.43)	0.49
E2*				
	**Cycle day 2**	35.51 ± 1.71 (34.80, 17.97)	35.02 ± 1.10 (34.76, 12.16)	0.65
	**Trigger day**	2135.17 ± 112.34 (2002.65, 907.76)	3383.61 ± 108.17 (2221.44, 1047.44)	0.03
*Data presented as Mean ± SD (interquartile range), independent samples *t* test, **Data presented as n (%), Mann-Whitney test. PCOS: Polycystic ovarian syndrome, OHSS: Ovarian hyperstimulation syndrome, BMI: Body mass index, FSH: Follicle-stimulating hormone, LH: Luteinizing hormone, E2: Estradiol				

**Table 2:** Comparison of ovarian stimulation characteristics between groups

**Parameters**	**PCOS without OHSS (n = 30)**	**PCOS with OHSS (n = 54)**	**P-value**
**Gonadotrophin dose (IU)**	1512.50 ± 50.87 (1425.00, 318.75)	1422.22 ± 33.11 (1425.00, 318.75)	0.25
**Duration of stimulation (days)**	14.20 ± 0.48 (14.5, 4.00)	12.56 ± 0.29 (12.00, 4.00)	0.005
**Number dominant follicle**	32.03 ± 1.23 (30.00, 2.50)	32.96 ± 0.77 (30.00, 10.00)	0.14
Data presented as Mean ± SD (interquartile range), Mann-Whitney test. PCOS: Polycystic ovarian syndrome, OHSS: Ovarian hyperstimulation syndrome			

**Table 3:** Comparison of oocyte parameters and fertilization rate between groups

**Parameters**	**PCOS without OHSS (n = 30)**	**PCOS with OHSS (n = 54)**	**P-value**
**Total number of oocytes**	19.00 ± 0.33 (20.00, 1.00)	25.24 ± 0.76 (23.00, 4.00)	0.001
**Number of MII**	13.20 ± 0.71 (14.00, 6.00)	17.85 ± 0.71 (18.5, 5.00)	0.0001
**Number of MI oocytes**	1.67 ± 0.33 (1.00, 3.00)	1.63 ± 0.23 (1.00, 3.00)	0.94
**Number of GV oocytes**	3.03 ± 0.43 (3.00, 3.00)	3.70 ± 0.53 (3.00, 4.00)	0.99
**Number of abnormal oocytes**	1.10 ± 0.26 (0.00, 2.00)	2.04 ± 0.56 (0.00, 2.00)	0.89 ${ߐ}^{\text{NS}}$
**Fertilization rate (%)**	72.52 ± 3.67 (67.55, 27.50)	72.25 ± 2.37 (73.21, 18.17)	0.29
**Zygote number 2PN**	9.40 ± 0.62 (9.50, 5.25)	12.85 ± 0.68 (14.00, 7.00)	0.036
Data presented as Mean ± SD (interquartile range), Mann-Whitney test. PCOS: Polycystic ovarian syndrome, OHSS: Ovarian hyperstimulation syndrome, MI: Metaphase I, MII: Metaphase II, GV: Germinal vesicle, 2PN: Two pronuclei			

**Table 4:** Comparison of embryo grading (day 3) between the studied groups

**Embryo grade**	**PCOS without OHSS (n = 30)**	**PCOS with OHSS (n = 54)**	**P-value**
**Grade I**	4.90 ± 0.56 (5.00, 4.25)	5.54 ± 0.50 (6.00, 6.00)	0.52
**Grade II**	2.70 ± 0.42 (3.00, 3.25)	3.13 ± 0.49 (2.00, 4.00)	0.94
**Grade III**	0.67 ± 0.15 (0.00, 1.00)	1.28 ± 0.28 (0.00, 2.00)	0.77
**Abnormal embryos**	0.70 ± 0.28 (0.00, 1.00)	0.46 ± 0.19 (0.00, 0.00)	0.08
**Arrested embryos**	1.90 ± 0.36 (1.00, 4.00)	1.39 ± 0.34 (0.00, 1.25)	0.017
Data presented as Mean ± SD (interquartile range), Mann-Whitney test. PCOS: Polycystic ovarian syndrome, OHSS: Ovarian hyperstimulation syndrome			

**Table 5:** Comparison of pregnancy rates in PCOS women with and without OHSS

**Pregnancy outcome**	**PCOS without OHSS (n = 30)**	**PCOS with OHSS (n = 54)**	**Total (n = 84)**	**P-value**
**Fresh embryo transfer **	9/18 (50%)	5/20 (25%)	14/38 (36.84%)	0.10
**Frozen embryo transfer**	3/12 (25%)	13/34 (38.24%)	16/46 (34.78%)	0.32
**Total pregnancy rate (n = 84)**	12/30 (40%)	18/54 (33.3%)	30/84 (35.7%)	0.54
Data presented as n (%), Chi-square tests. PCOS: Polycystic ovarian syndrome, OHSS: Ovarian hyperstimulation syndrome				

## 4. Discussion

ART has gained worldwide acceptance as an accepted treatment for infertility. Despite recent advances in in vitro fertilization and ICSI, only one-third of these treatments result in a live birth (21).

One of the common iatrogenic complications that may result from ovulation hyperstimulation is OHSS, which has serious effects on the physical and mental health of infertile women and may even increase the complications in the perinatal period of pregnant women (22). Since follicular growth is affected by PCOS, especially after controlled ovarian hyperstimulation, a decreased number of good quality oocytes/embryos in ART cycles were found to be a common problem (8).

The results of the present study were comparable between the 2 groups regarding age, BMI, and duration of infertility (Table I). These findings were essential to ensure statistical matching and reduce any variations that could affect the study's outcome. Primary infertility was significantly higher in both groups and higher in PCOS with OHSS than in the other group. This result is in accordance with a study by Jabeen et al. who concluded that as compared to secondary infertility, primary infertility's superiority is higher, which may be attributed to increasing marriage age, which aggravates reasons with male variables and unexplained infertility (23).

Regarding hormonal levels (basal FSH, LH, and E2), no statistical difference was found (Table I) as both groups were within the same age limits and both were diagnosed with PCOS. Only the E2 level on trigger day was significantly higher in the OHSS group, and this is logical as it is well known that a rapid rise in serum E2 concentrations > 2500 pg/mL is an important predictive factor of OHSS due to the large number of growing follicles on the day of triggering ( > 14 follicles with a diameter of 11 mm) (24). However, the duration of stimulation was shorter in the OHSS group (Table III), and this is related to high ovarian reserve with significantly lower oocyte yield in the non-OHSS group, which agrees with the results of previous studies that stated that in women with PCOS, although the number of retrieved and mature oocytes was decreased in prolonged stimulations, clinical and ongoing pregnancy rates were not affected (25). In contrast, others did not agree with the present result and reported that “duration of gonadotropin stimulation is associated with follicular growth, oocyte maturity, quality, and endometrial development. Insufficient period of gonadotropin exposure may lead to nuclear or cytoplasmic immaturity of oocytes. Moreover, prolonged stimulation duration may cause post-maturity of oocytes or apoptosis of granulosa cells and oocytes. It may also result in elevated progesterone (P4) and E2 levels and impaired endometrial receptivity (26, 27)”. The results of the current study recorded a higher number of oocytes retrieved, mature oocytes, and the number of pronuclei in the OHSS group (Table II) but did not show any significant difference in embryo quality (Table IV) or pregnancy rate following fresh or frozen embryo transfer (Table V). These findings were in agreement with previous studies, which reported that in women developing OHSS, the mean number of oocytes retrieved was significantly higher. However, embryo quality and pregnancy rate did not differ between groups, and this may be attributed to the lower oocyte quality. The fertilization rate was compensated by the larger number of oocytes retrieved (28). The difficulties faced by the study were the limited number of cases due to incomplete data available.

## 5. Conclusion

From the results of the present study; we concluded that women with PCOS who have a higher chance of developing OHSS, may result in higher oocyte yields and higher number of mature oocytes, which may compensate for the adverse effects of OHSS on embryo quality or pregnancy rate; therefore, no significant difference was observed neither in the number of good quality embryo nor in pregnancy rate whether fresh or frozen transferred cycles.

##  Data Availability

Data supporting the findings of this study are available upon reasonable request from the corresponding author.

##  Author Contributions

MA. Jwad: Conceptualization and data curation. MH. Khaleefah: Formal analysis and data curation. R. Abd AlFattah Naser: Investigation.

##  Conflict of Interest

The authors declare that there is no conflict of Interest.
